# Comparison of Standalone Tanito Microhook Trabeculotomy Between Unilateral and Bilateral Incision Groups

**DOI:** 10.3390/jcm14061976

**Published:** 2025-03-14

**Authors:** Kazunobu Sugihara, Chisako Ida, Hinako Ohtani, Masaki Tanito

**Affiliations:** Department of Ophthalmology, Shimane University Faculty of Medicine, 89-1 Enya, Izumo 693-8501, Japan; ksugi@med.shimane-u.ac.jp (K.S.); chisako212@med.shimane-u.ac.jp (C.I.); hinakootani0208@med.shimane-u.ac.jp (H.O.)

**Keywords:** microhook ab interno trabeculotomy, goniotomy, Tanito microhook (TMH), intraocular pressure, minimally invasive glaucoma surgery, surgical complication, hyphema

## Abstract

**Background/Objectives:** Trabeculotomy using the Tanito microhook (TMH) is a minimally invasive glaucoma surgery (MIGS) technique that effectively reduces intraocular pressure (IOP). The optimal incision extent for standalone TMH remains unclear. This study aimed to compare the surgical efficacy and safety of unilateral (120-degree) and bilateral (240-degree) incisions in standalone TMH for primary open-angle glaucoma or late-onset juvenile open-angle glaucoma in patients without a history of intraocular surgery. **Methods:** This retrospective study analyzed 81 eyes of 48 patients who underwent standalone TMH at Shimane University Hospital. Patients were categorized into unilateral (29 eyes) or bilateral (52 eyes) incision groups. The primary outcomes were IOP reduction and medication score changes over 12 months. Secondary outcomes included best-corrected visual acuity (BCVA), anterior chamber (AC) flare, corneal endothelial cell density (CECD), and postoperative complications, particularly hyphema, assessed using the Shimane University Postoperative Hyphema Scoring System (SU-RLC). Multivariate mixed-effects regression and Kaplan–Meier survival analyses were performed. **Results:** At 12 months, IOP reduction was comparable between the unilateral (23%) and bilateral (28%) groups (*p* = 0.29). The unilateral group had a significantly lower medication score at postoperative day 3 (*p* = 0.0057) and week 2 (*p* = 0.033). No significant differences were observed in BCVA, AC flare, CECD, or visual field mean deviation (MD). However, the bilateral group had significantly higher hyphema scores (*p* = 0.017), with more cases of layered hyphema. **Conclusions:** Unilateral standalone TMH achieved equivalent IOP and medication score reductions compared to bilateral incisions, with a lower risk of early postoperative hyphema. The unilateral approach may be preferable for faster visual recovery.

## 1. Introduction

Glaucoma is a progressive neurodegenerative eye disease that results in optic nerve damage, visual field loss, and eventual vision impairment [1,2]. Elevated intraocular pressure (IOP) remains a primary risk factor for disease progression, and the only established treatment strategy involves reducing IOP through pharmacological therapy, laser interventions, or surgical procedures. Among the various surgical options, minimally invasive glaucoma surgery (MIGS) has gained popularity due to its balance between efficacy and safety [3,4,5]. One such MIGS technique, the Tanito microhook ab interno trabeculotomy (TMH), employs a metal hook to incise the trabecular meshwork and has been shown to effectively lower IOP while reducing the dependence on antiglaucoma medications [6].

Initially, TMH was performed by creating incisions in both the nasal and temporal trabecular meshwork, achieving a total incision angle of approximately 240 degrees. Previous research on cadaveric eyes suggested that the extent of trabeculotomy may influence postoperative IOP reduction [7]. While some clinical studies have reported a correlation between surgical extent and the magnitude of IOP reduction [8,9,10], others have found no significant difference [11,12,13,14,15,16]. Many of these studies compared different surgical techniques or included patient populations with varying preoperative IOP levels and baseline characteristics, which may introduce bias [8,9,10,13,16]. Additionally, some studies were limited by small sample sizes [11,14].

While the relationship between cataract extraction and IOP reduction remains inconclusive [6,17,18], some studies suggest that cataract removal at the time of trabeculotomy/goniotomy may contribute to greater IOP reduction [19]. This highlights the challenge of assessing the isolated effect of trabecular meshwork incision when cataract surgery is performed simultaneously. In a previous study, we compared the surgical efficacy and safety of 120-degree versus 240-degree TMH in eyes undergoing combined TMH and cataract surgery [20]. The results suggested that IOP reduction following TMH was comparable between unilateral and bilateral incisions. However, in MIGS procedures, the additional IOP-lowering effect of cataract surgery must be considered [21,22,23,24,25]. Thus, an ideal approach to evaluating the impact of incision extent in trabeculotomy should focus solely on standalone trabeculotomy cases.

In the present study, we conducted a comprehensive comparison of surgical outcomes between unilateral (approximately 120-degree) and bilateral (approximately 240-degree) incisions in standalone TMH. To minimize confounding variables, we included only patients with open-angle glaucoma who had no prior history of intraocular surgery.

## 2. Subjects and Methods

### 2.1. Subjects

This research was conducted in accordance with the principles outlined in the Declaration of Helsinki and received approval from the institutional review board (IRB) of Shimane University Hospital (IRB No. 20200517-1, revised protocol issued on 22 July 2024). Written informed consent for the surgical procedure was obtained from all participants prior to surgery. However, individual written consent for publication was not mandated by the IRB; instead, the study protocol was publicly posted within the study institutions to inform participants. Only de-identified data were used for statistical analysis.

A retrospective review identified eligible participants based on the following inclusion criteria: individuals who underwent surgery at Shimane University Hospital between April 2018 and August 2023, performed by one of two surgeons (K.S. or M.T.); those who underwent trabeculotomy using a microhook (TMH) as the sole surgical procedure; individuals aged 18 years or older; and those diagnosed with primary open-angle glaucoma (POAG) or late-onset juvenile open-angle glaucoma (JOAG) without any history of prior intraocular or glaucoma surgeries, with a minimum postoperative follow-up of 12 months. No explicit exclusion criteria were established. A total of 81 eyes from 48 participants were identified in our institutional database. At our institution, bilateral incisions were the predominant approach until 2019, whereas from 2021 onward, a nasal-only incision became the preferred technique. This transition allowed for the collection of data on both unilateral and bilateral incision approaches, with 29 eyes from 17 participants categorized into the unilateral group and 52 eyes from 31 participants assigned to the bilateral group for comparative analysis.

### 2.2. Parameters

Clinical parameters were extracted from medical records, including age, sex, glaucoma classification, surgical approach, preoperative and postoperative best-corrected visual acuity (BCVA), intraocular pressure (IOP), number of antiglaucoma medications, anterior chamber (AC) flare (measured using the FM-600 laser flare meter, Kowa, Nagoya, Japan), corneal endothelial cell density (CECD) (measured with the EM-3000 specular microscope, Tomey, Nagoya, Japan), axial length (measured using the OA-2000 optical biometer, Tomey, Nagoya, Japan), visual field mean deviation (MD) (central 30-2 program, Humphrey Visual Field Analyzer, Carl Zeiss Meditec, Dublin, CA, USA), and postoperative follow-up duration. Decimal BCVA values were converted to the logarithm of the minimum angle of resolution (logMAR) VA. The following values were assigned for low-vision states: counting fingers (0.0025), hand motions (0.002), light perception (0.0016), and no light perception (0.0013) [26]. IOP was measured using Goldmann applanation tonometry, except on postoperative day 3, when the iCARE rebound tonometer (M.E. Technica, Tokyo, Japan) was used. The medication score was calculated by assigning one point per topical medication component or per tablet of oral acetazolamide.

Details regarding the trabeculotomy site, perioperative complications, complication management, and additional glaucoma surgeries were extracted from surgical and medical records. Postoperative time points were categorized as follows: postoperative day (POD) 3 (1–3 days), postoperative week (POW) 2 (1–3 weeks), postoperative month (POM) 3 (2–4 months), POM 6 (5–7 months), POM 9 (8–10 months), and POM 12 (11–14 months).

The Shimane University Postoperative Hyphema Scoring System (SU-RLC) [27] was utilized to quantify hyphema severity. This system employs slit-lamp examination to assess three components: red blood cells (RBCs) (R), layering (L), and clot formation (C). RBCs are graded from 0 (no visible floating RBCs in the AC) to 3 (dense floating RBCs obscuring the iris pattern). Layering is scored from 0 (absent) to 3 (layering above the lower pupillary margin). Clot formation is rated as 0 (absent) or 1 (present). The cumulative SU-RLC score ranges from 0 to 7, providing an overall assessment of hyphema severity. The highest SU-RLC score observed during follow-up was used for analysis, with most cases reaching peak severity within the first three postoperative days.

### 2.3. Surgical Techniques

The surgical approach has been described in detail in previous work [6,28]. In brief, TMH was performed using specifically designed spatula-shaped microhooks (M-2215S, 2215R, and 2215L, Inami, Tokyo, Japan). Viscoelastic material (1% sodium hyaluronate or Opegan Hi) was introduced into the AC through corneal ports created with a 20-gauge microvitreoretinal knife at the 2–3 o’clock and 9–10 o’clock positions. A microhook was inserted into the AC via a corneal port, and the opposite angle was visualized using a Swan-Jacob gonioprism lens. The microhook tip was inserted into Schlemm’s canal and advanced circumferentially to incise the inner wall of Schlemm’s canal and trabecular meshwork for at least three hours, either at the nasal site or at both nasal and temporal locations. Following TMH, viscoelastic material was removed, and corneal ports were sealed by stromal hydration. At the conclusion of surgery, a subconjunctival injection of 2 mg of betamethasone sodium phosphate was administered, and 0.3% ofloxacin ointment was applied. Postoperatively, topical 1.5% levofloxacin and 0.1% betamethasone were prescribed four times daily for 3–4 weeks.

### 2.4. Statistical Tests

Comparative analyses between the unilateral and bilateral incision groups were conducted using unpaired *t*-tests for continuous variables, Fisher’s exact test for categorical variables, and the Cochran–Armitage trend test for ordinal data. To address potential biases due to age and sex differences among the subjects, as well as the inclusion of bilateral eyes from individual participants, mixed-effects regression analyses were performed to evaluate the impact of the surgical approach on IOP, medication score, BCVA, AC flare, CECD, and MD.

Kaplan–Meier survival analysis was conducted to estimate the probability of maintaining controlled IOP. Successful IOP control was assessed by defining failure based on multiple criteria: IOP exceeding 18 mmHg (criterion A, D), 15 mmHg (criterion B, E), or 12 mmHg (criterion C, F) after three months postoperatively; an IOP reduction of less than 20% (criteria A–C) or 0% (criteria D–F) after three months postoperatively; the need for additional glaucoma surgery at any point; or loss of light perception. Cases that did not meet the failure criteria were considered to have survived and treated as censored. Since prolonged hypotony was not observed, failure related to hypotony was not included in the survival analysis definition. Antiglaucoma medication use was not factored into survival curve analysis, as most patients continued medication postoperatively. Log-rank tests were employed to compare survival distributions between the surgical groups. Cox proportional hazards regression models were used to identify factors associated with IOP control under each criterion (A–F).

Missing values were imputed using the last observation carried forward (LOCF) method. If no prior observation was available, the next observation carried backward (NOCB) method was applied. For cases requiring additional glaucoma surgery, postoperative IOP and medication scores were considered missing beyond the surgical intervention, while other parameters were included for the full follow-up period. A *p*-value of <0.05 was deemed statistically significant. Statistical analyses were performed using JMP Pro version 17.2 (SAS Institute, Inc., Cary, NC, USA).

## 3. Results

The baseline characteristics of the study participants, including age, sex, laterality, glaucoma type, BCVA, IOP, medication score, axial length, AC flare, CECD, and preoperative MD values, are summarized in Table 1. The mean ages in the unilateral and bilateral incision groups were 47.5 and 50.9 years, respectively, with males comprising 41% and 68% of each group. The total trabecular meshwork incision width averaged 3.6 clock hours in the unilateral group and 7.1 clock hours in the bilateral group. Apart from this, no significant differences were observed in any other demographic parameters between the two surgical groups.

In this study, data from both eyes were included in the analysis for cases in which bilateral surgery was performed to ensure an adequate sample size. To adjust for potential bias introduced by selecting both eyes from the same patient, a mixed-effects model was applied. This model also accounted for age and sex as covariates. The results of the analysis assessing the impact of incision extent on various parameters are presented in Table 2. IOP, medication score, and AC flare demonstrated significant associations with postoperative duration, indicating that these factors were influenced by the surgical intervention. In contrast, no significant association was found between incision extent (i.e., unilateral or bilateral incision) and IOP, medication score, BCVA, CECD, or MD. Additionally, no significant interaction was detected between postoperative duration and incision extent for any of these parameters.

Table 3 presents a comparison of IOP between the unilateral and bilateral incision groups. The preoperative IOP, which was 22.3 mmHg in the unilateral group and 22.1 mmHg in the bilateral group, decreased to 17.1 mmHg (a 23% reduction) in the unilateral group and 16.0 mmHg (a 28% reduction) in the bilateral group at 12 months postoperatively. At the 3-month postoperative mark, the bilateral group had a significantly lower IOP than the unilateral group. However, at all other time points up to 12 months, no significant difference in IOP was observed between the two groups.

Table 4 presents a comparison of the number of antiglaucoma medications between the unilateral and bilateral incision groups. The preoperative medication score, which was 3.9 in the unilateral group and 3.6 in the bilateral group, decreased to 2.8 (a 28% reduction) and 3.0 (a 17% reduction), respectively, at 12 months postoperatively. At postoperative day 3 and week 2, the unilateral group had a significantly lower medication score than the bilateral group. However, at all other time points up to 12 months, no significant difference in medication scores was found between the two groups.

Table 5 presents a comparison of postoperative complications, excluding hyphema, and the required interventions between the unilateral and bilateral incision groups. There was no significant difference between the groups regarding the incidence of complications or the necessity for interventions. All eight additional glaucoma surgeries recorded in this dataset were trabeculectomies.

Table 6 provides a comparison of postoperative hyphema scores between the two groups. The overall incidence of hyphema, including cases with minimal floating RBCs in the AC, was 93% in the unilateral incision group and 96% in the bilateral incision group. The mean total RLC score was 2.6 in the unilateral incision group and 3.6 in the bilateral incision group, demonstrating a statistically significant difference (*p* = 0.0017). While the majority of cases in the unilateral incision group had a score of 3 or lower, 51% of cases in the bilateral incision group had a score of 4 or higher.

For individual components of the hyphema score, both the R and L scores were significantly higher in the bilateral incision group compared to the unilateral incision group (*p* = 0.039 and *p* = 0.0013, respectively), whereas the C score showed no significant difference between the groups. The incidence of layered hyphema (L score ≥ 1) was 28% in the unilateral incision group and 61% in the bilateral incision group.

Table 7 presents a comparison of BCVA between the unilateral and bilateral incision groups. The preoperative BCVA was −0.02 in the unilateral incision group and 0.03 in the bilateral incision group, remaining unchanged at −0.03 and −0.01, respectively, at 12 months postoperatively. BCVA remained comparable between the two groups throughout the 12-month study period. The lack of changes in BCVA suggests that a visually significant cataract did not develop in most cases in either group.

Table 8 presents a comparison of AC flare between the unilateral and bilateral incision groups. The preoperative AC flare was 9.7 pc/ms in the unilateral group and 8.1 pc/ms in the bilateral group, showing minimal change at 12 months postoperatively, with values of 8.0 pc/ms and 8.5 pc/ms, respectively. AC flare remained similar between the groups throughout the study period.

Table 9 presents a comparison of CECD between the unilateral and bilateral incision groups. The preoperative CECD was 2601 cells/mm^2^ in the unilateral group and 2572 cells/mm^2^ in the bilateral group, with minor variations at 12 months postoperatively, measuring 2512 cells/mm^2^ and 2553 cells/mm^2^, respectively. CECD remained equivalent between the two groups over the 12-month period.

Table 10 presents a comparison of visual field MD between the unilateral and bilateral incision groups. The preoperative MD was −9.6 dB in the unilateral group and −8.7 dB in the bilateral group, with slight changes at 12 months postoperatively, recorded as −9.3 dB and −9.1 dB, respectively. MD values were similar between the two groups both preoperatively and at 12 months postoperatively.

**Figure 1:**
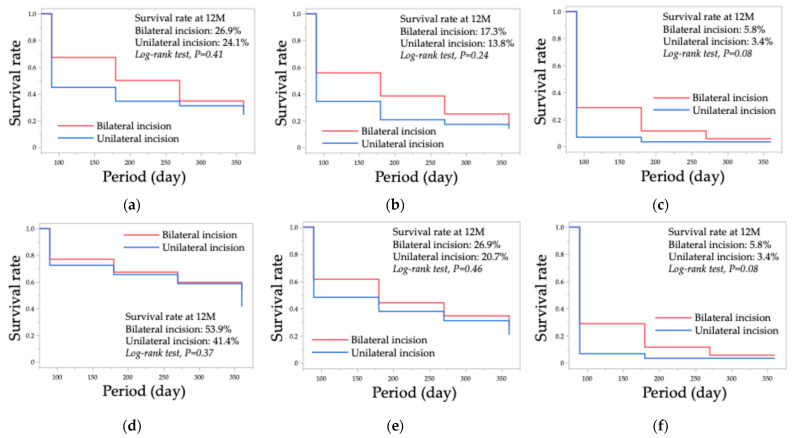
Kaplan–Meier survival curves illustrate IOP control outcomes for the unilateral and bilateral incision groups based on six failure criteria. Criterion A (**a**) defines failure as an IOP reduction of less than 20% from preoperative levels and/or an IOP exceeding 18 mmHg. Criterion B (**b**) applies the same percentage reduction threshold but with an upper IOP limit of 15 mmHg, while Criterion C (**c**) further lowers this threshold to 12 mmHg. Criterion D (**d**) considers failure to have occurred when the IOP reduction is less than 0% from preoperative levels and/or exceeds 18 mmHg. Similarly, Criterion E (**e**) sets this limit at 15 mmHg, and Criterion F (**f**) at 12 mmHg. Additionally, patients who required further glaucoma surgery or lost light perception were classified as failures.

Figure 1 displays the Kaplan–Meier survival curves for successful IOP control in both the unilateral and bilateral incision groups. Failure was defined using three criteria: IOP exceeding 18 mmHg (Criterion A), 15 mmHg (Criterion B), or 12 mmHg (Criterion C), in combination with an IOP reduction of less than 20% from the preoperative level. At 12 months postoperatively, the cumulative survival rates for successful IOP control were 24.1% in the unilateral incision group and 26.9% in the bilateral incision group based on Criterion A; 13.8% and 17.3%, respectively, based on Criterion B; and 3.4% and 5.8%, respectively, based on Criterion C. The log-rank test yielded *p*-values of 0.41 for Criterion A, 0.24 for Criterion B, and 0.08 for Criterion C, indicating no significant differences in survival rates between the unilateral and bilateral incision groups.

Unlike filtering surgeries, where a reduction of less than 20% in IOP may be a meaningful indicator of failure, applying this criterion to trabeculotomy might be overly stringent [28]. Therefore, an additional analysis was conducted using alternative failure criteria, where failure was defined as IOP exceeding 18 mmHg (Criterion D), 15 mmHg (Criterion E), or 12 mmHg (Criterion F), along with any postoperative IOP exceeding the preoperative level. Under these criteria, at 12 months postoperatively, the cumulative survival rates for successful IOP control were 41.4% in the unilateral incision group and 53.9% in the bilateral incision group based on Criterion D, 20.7% and 26.9%, respectively, based on Criterion E, and 3.4% and 5.8%, respectively, based on Criterion F. The log-rank test yielded *p*-values of 0.37 for Criterion D, 0.46 for Criterion E, and 0.08 for Criterion F, again showing no significant differences in survival rates between the unilateral and bilateral incision groups.

Finally, Table 11 presents the results of the Cox proportional hazards model analysis, which evaluated factors potentially influencing IOP control based on each of the six failure criteria (A–F). In a model adjusted for age, sex, glaucoma type, preoperative IOP, preoperative medication score, and the presence of surgical complications, the incision extent was not identified as a significant factor under any of the criteria.

For Criterion D, the presence of surgical complications showed a borderline significant association with surgical success (*p* = 0.048). For Criterion E, a higher preoperative IOP was significantly associated with surgical failure (*p* = 0.0028).

## 4. Discussion

This study is a retrospective observational study comparing the surgical efficacy and safety of standalone TMH between the unilateral TM incision group and the bilateral TM incision group. Regarding surgical efficacy parameters, including IOP, medication score, visual field MD, and survival analysis, no significant differences were observed between the two groups, except for a few instances. Additionally, safety evaluation parameters, including BCVA, AC flare, and CECD, showed no differences between the groups. The only clinically remarkable difference observed was in postoperative hyphema, where the bilateral group had a significantly higher hyphema score than the unilateral group.

A study using perfused autopsy eyes suggested that the extent of trabeculotomy may influence IOP reduction [7]. Investigating the impact of incisions on one or both sides and examining the correlation between incision extent and IOP reduction following goniotomy or trabeculotomy may provide further insights. Previous studies have compared the surgical effects of different incision extents in trabeculotomy/goniotomy; however, these studies included cases with simultaneous cataract surgery, pseudophakic eyes, or both [11,14,16,20]. As a result, the pure effect of MIGS could not be fully assessed. A key strength of our study is that we focused on patients with phakic POAG, including adult-onset JOAG, who had no prior ocular surgeries, allowing us to compare different incision extents in TMH more directly.

In this study, univariate analysis showed that at three months postoperatively, IOP was significantly lower in the bilateral group (Table 3). However, three days and three months postoperatively, the medication score was significantly lower in the unilateral group (Table 4). Additionally, survival analysis revealed no significant differences between the two groups (Figure 1). In multivariate analysis, no significant differences in IOP or medication score were found between the groups, but duration was a significant factor (Table 2). These findings indicate that both groups experienced significant postoperative reductions in IOP and medication scores regardless of incision extent. A previous study compared nasal and bilateral TMH incisions combined with cataract surgery in patients with open-angle glaucoma and found no significant differences in IOP or medication score between the groups throughout the study period [20]. Similarly, other studies on TMH reported no significant differences in IOP reduction, medication score, or success rates between 1-quadrant and 2-quadrant TMH procedures [14]. In studies on other trabeculotomy/goniotomy techniques, including Trabectome surgery with 0- to 160-degree incisions [29] and gonioscopy-assisted transluminal trabeculotomy (GATT) with 150- to 320-degree incisions [11], no significant differences in surgical outcomes were observed. A multicenter study also found no significant differences in IOP reduction, medication number, or cumulative survival probability for complete and qualified success rates among 120-, 240-, and 360-degree standalone goniotomy procedures, as well as when these were combined with cataract surgery [16]. Given these findings, along with our current results, unilateral trabeculotomy may be sufficient for most cases of open-angle glaucoma. However, individual variability may exist, highlighting the need for further research to determine specific cases where a more extensive trabecular meshwork incision could be more beneficial.

Regarding postoperative complications and additional procedures, no significant differences were found between the unilateral and bilateral groups. However, postoperative hyphema was notably more severe in the bilateral group. This finding aligns with our previous analysis of TMH combined with cataract surgery [20]. A greater incidence of postoperative layered hyphema was reported in GATT with a 360-degree incision compared to a 180-degree incision [12]. Similarly, goniotomy with a 360-degree incision resulted in more frequent hyphema than that with a 120-degree incision [15,16]. Previous research also demonstrated that early postoperative hyphema scores were higher in eyes undergoing TMH compared to those receiving first- and second-generation iStent implants [27,30]. These findings suggest that hyphema severity correlates with incision width in angle surgeries. Furthermore, clot formation following trabeculotomy has been identified as a significant factor contributing to elevated IOP one week postoperatively [31]. Another study indicated that a two-quadrant TMH procedure had a significantly higher proportion of transient postoperative IOP elevation [14]. In the present study, multivariate analysis revealed a significant interaction between medication score, duration, and incision width (Table 1), with a lower early postoperative medication score in the unilateral group compared to the bilateral group (Table 4). Therefore, differences in the degree of anterior chamber bleeding may explain the variations in early postoperative medication scores. However, despite early postoperative differences in hyphema scores, no significant impact was observed on other safety parameters or final visual function, though these differences may have influenced early visual recovery. Given that IOP reduction was comparable between the two groups, the unilateral incision approach, which may facilitate a faster visual recovery, could be clinically more advantageous than the bilateral incision method.

The study had several limitations, including its retrospective design, differences in sample size between the groups, relatively small cohort, and the inclusion of both eyes from some subjects. However, the use of mixed regression analysis likely mitigated some biases. Additionally, the lack of information regarding anticoagulant and antiplatelet agent use was a limitation. Another limitation was the relatively short follow-up period. Nonetheless, the study had strengths, including a comprehensive assessment of patient clinical characteristics and the inclusion of only patients without a history of intraocular surgery.

## 5. Conclusions

In conclusion, our study demonstrated that the extent of IOP and medication number reductions achieved with standalone TMH were equivalent regardless of incision width. While the overall safety profiles were similar, procedures with a wider incision width resulted in increased hyphema severity in the early postoperative period. When performing TMH on phakic eyes with POAG/JOAG without a history of prior surgery, a trabecular meshwork incision of approximately one-third of the circumference results in the maximum clinical effect.

## Figures and Tables

**Table 1:** Baseline characteristics of study participants.

Parameters	Unilateral Incision	Bilateral Incision	*p*-Value
N, subjects	17	31	
Age, years			
Mean ± SD	47.5 ± 12.3	50.9 ± 9.4	0.29
95% CI	69.2, 72.7	70.0, 73.2	
Sex, n (%)			
Male	7 (41)	21 (68)	0.13
Female	10 (59)	10 (32)	
N, eyes	29	52	
Incision extent, clock hours			
Nasal	3.6 ± 0.5	3.6 ± 0.5	0.61
Temporal		3.5 ± 0.5	
Total	3.6 ± 0.5	7.1 ± 0.4	<0.0001 **
Laterarity, n (%)			
Right eye	13 (45)	27 (52)	0.64
Left eye	16 (55)	25 (48)	
Glaucoma type, n (%)			
POAG	22 (76)	38 (73)	>0.99
JOAG	7 (24)	14 (27)	
BCVA, LogMAR			
Mean ± SD	−0.02 ± 0.10	0.03 ± 0.22	0.21
95% CI	−0.05, 0.01	−0.03, 0.10	
IOP, mmHg			
Mean ± SD	22.3 ± 9.0	22.1 ± 8.0	0.90
95% CI	18.9, 25.8	19.9, 24.3	
Medication score			
Mean ± SD	3.9 ± 1.1	3.6 ± 0.8	0.23
95% CI	3.5, 4.3	3.4, 3.9	
Axial length, mm			
Mean ± SD	26.1 ± 2.0	25.9 ± 1.8	0.60
95% CI	24.3, 25.0	25.4, 26.4	
AC flare, pc/ms			
Mean ± SD	9.7 ± 9.3	8.1 ± 4.6	0.31
95% CI	6.1, 13.2	6.8, 9.4	
CECD, cells/mm^2^			
Mean ± SD	2601 ± 268	2572 ± 213	0.59
95% CI	2499, 2703	2512, 2631	
MD, dB			
Mean ± SD	−9.6 ± 8.2	−8.7 ± 7.3	0.61
95% CI	−6.5, −12.7	−6.6, −10.7	

*p*-values are determined using an unpaired *t*-test for continuous variables and Fisher’s exact test for categorical variables. A ** denotes statistical significance at *p* < 0.01. Abbreviations: SD—standard deviation; CI—confidence interval; POAG—primary open-angle glaucoma; JOAG—juvenile open-angle glaucoma; BCVA—best-corrected visual acuity; IOP—intraocular pressure; AC—anterior chamber; pc/ms—photon counts per millisecond; CECD—corneal endothelial cell density; MD—mean deviation of visual field; dB—decibels.

**Table 2:** Adjustment of demographic factor differences using mixed-effects regression analysis.

Parameters	F Value	*p*-Value
IOP		
Age	1.6	0.21
Sex	0.7	0.42
Unilateral/Bilateral	0.4	0.51
Postoperative duration	12.0	<0.0001 **
Unilateral/Bilateral × Postoperative duration	1.9	0.072
Medication score		
Age	7.0	0.011 *
Sex	1.3	0.26
Unilateral/Bilateral	1.9	0.17
Postoperative duration	23.5	<0.0001 **
Unilateral/Bilateral × Postoperative duration	4.1	0.0002 **
BCVA, LogMAR		
Age	2.5	0.12
Sex	0.4	0.52
Unilateral/Bilateral	1.0	0.33
Postoperative duration	1.0	0.41
Unilateral/Bilateral × Postoperative duration	0.2	0.98
AC flare, pc/ms		
Age	0.4	0.55
Sex	0.5	0.49
Unilateral/Bilateral	0.0	0.94
Postoperative duration	2.2	0.045 *
Unilateral/Bilateral × Postoperative duration	1.9	0.087
CECD, cells/mm^2^		
Age	1.4	0.24
Sex	2.2	0.14
Unilateral/Bilateral	0.0	0.97
Postoperative duration	2.6	0.081
Unilateral/Bilateral × Postoperative duration	0.8	0.45
MD, dB		
Age	4.4	0.041 *
Sex	0.3	0.59
Unilateral/Bilateral	0.0	0.97
Postoperative duration	0.1	0.81
Unilateral/Bilateral × Postoperative duration	0.1	0.75

*p*-values are calculated using a mixed-effects regression model to adjust for biases arising from differences in age, sex distribution, and the inclusion of bilateral eyes from the same subject. The * and ** indicate *p* < 0.05 and *p* < 0.01, respectively. Abbreviations: IOP—intraocular pressure; BCVA—best-corrected visual acuity; LogMAR—logarithm of the minimum angle of resolution; AC—anterior chamber; pc/ms—photon counts per millisecond; CECD—corneal endothelial cell density; MD—visual field mean deviation; dB—decibels.

**Table 3:** Comparison of preoperative and postoperative iop between the unilateral and bilateral incision groups.

Parameters	Unilateral Incision	Bilateral Incision	*p*-Value
Preoperative			
Mean ± SD, mmHg	22.3 ± 9.0	22.1 ± 8.0	0.90
95% CI	18.9, 25.8	19.9, 24.3	
POD3			
Mean ± SD	13.1 ± 9.4	15.9 ± 9.5	0.21
95% CI	9.5, 16.7	13.2, 18.5	
POW2			
Mean ± SD	18.1 ± 6.7	18.1 ± 8.3	>0.99
95% CI	15.5, 20.6	15.8, 20.4	
POM1			
Mean ± SD	17.9 ± 6.6	16.2 ± 4.9	0.20
95% CI	15.4, 20.4	14.8, 17.6	
POM3			
Mean ± SD	17.9 ± 8.4	14.7 ± 3.9	0.027 *
95% CI	14.7, 21.1	13.6, 15.8	
POM6			
Mean ± SD	16.5 ± 5.6	15.5 ± 4.1	0.38
95% CI	14.3, 18.7	14.3, 16.7	
POM9			
Mean ± SD	16.4 ± 3.3	15.3 ± 3.8	0.22
95% CI	15.0, 17.8	14.2, 16.4	
POM12			
Mean ± SD	17.1 ± 5.0	16.0 ± 3.7	0.29
95% CI	15.0, 19.2	14.9 ± 17.0	

*p*-values are calculated using an unpaired *t*-test. The * indicates *p* < 0.05. Abbreviations: SD—standard deviation; CI—confidence interval; POD—postoperative day; POW—postoperative week; POM—postoperative month.

**Table 4:** Comparison of preoperative and postoperative medication score between the unilateral and bilateral incision groups.

Parameters	Unilateral Incision	Bilateral Incision	*p*-Value
Preoperative			
Mean ± SD	3.9 ± 1.1	3.6 ± 0.8	0.23
95% CI	3.5, 4.3	3.4, 3.9	
POD3			
Mean ± SD	2.2 ± 1.0	2.9 ± 1.0	0.0057 **
95% CI	1.8, 2.6	2.6, 3.1	
POW2			
Mean ± SD	2.3 ± 1.3	3.0 ± 1.2	0.033 *
95% CI	1.9, 2.8	2.6, 3.3	
POM1			
Mean ± SD	2.4 ± 1.2	3.0 ± 1.3	0.052
95% CI	2.0, 2.9	2.7, 3.4	
POM3			
Mean ± SD	2.7 ± 1.2	2.8 ± 0.8	0.70
95% CI	2.2, 3.2	2.5, 3.0	
POM6			
Mean ± SD	2.6 ± 0.9	2.9 ± 0.9	0.29
95% CI	2.3, 3.0	2.6, 3.1	
POM9			
Mean ± SD	2.7 ± 0.8	3.0 ± 0.8	0.17
95% CI	2.4, 3.1	2.8, 3.2	
POM12			
Mean ± SD	2.8 ± 0.9	3.0 ± 0.8	0.20
95% CI	2.4, 3.1	2.8, 3.3	

*p*-values are calculated using an unpaired *t*-test. The * and ** indicate *p* < 0.05 and *p* < 0.01, respectively. Abbreviations: SD—standard deviation; CI—confidence interval; POD—postoperative day; POW—postoperative week; POM—postoperative month.

**Table 5:** Postoperative complications (except for hyphema) and interventions.

Parameters	Unilateral Incision	Bilateral Incision	*p*-Value
IOP spike (>30 mmHg)	3 (10)	11 (21)	0.18
Fibrin formation	0 (0)	3 (6)	0.55
Additional glaucoma surgery	5 (17)	3 (6)	0.13
Hyphema washout	1 (3)	3 (6)	>0.99
AC paracenthesis	0 (0)	1 (2)	>0.99

*p*-values are calculated using Fisher’s exact probability test. Abbreviations: IOP—intraocular pressure; AC—anterior chamber.

**Table 6:** Postoperative hyphema severity scored by SU-RLC scoring system.

Parameters	Unilateral Incision	Bilateral Incision	*p*-Value
Any hyphema, n (%)	27 (93)	50 (96)	0.61
Total RLC score			
Mean ± SD	2.6 ± 1.5	3.6 ± 1.9	0.017 *
95% CI	2.0, 3.1	3.0, 4.1	
0, n (%)	2 (7)	2 (4)	0.017 *
1	5 (17)	6 (12)	
2	8 (28)	10 (19)	
3	9 (31)	8 (15)	
4	1 (3)	8 (15)	
5	2 (7)	7 (13)	
6	2 (7)	8 (15)	
7	0 (0)	3 (6)	
R score			
0, n (%)	3 (10)	2 (4)	0.039 *
1	12 (41)	16 (31)	
2	9 (31)	14 (27)	
3	5 (17)	20 (38)	
L score			
0, n (%)	21 (72)	19 (39)	0.0013 **
1	6 (21)	17 (33)	
2	2 (7)	13 (25)	
3	0 (0)	3 (6)	
C score			
0, n (%)	10 (34)	22 (42)	0.49
1	19 (66)	30 (58)	

*p*-values are calculated using an unpaired *t*-test for continuous data and the Cochran–Armitage trend test for ordinal data. The * and ** indicate *p* < 0.05 and *p* < 0.01, respectively. Abbreviations: SU-RLC—Shimane University postoperative hyphema scoring system; R score—score for floating red blood cell density in the anterior chamber; L score—score for layered hyphema; C score—score for blood clot in the anterior chamber; SD—standard deviation; CI—confidence interval.

**Table 7:** Comparison of preoperative and postoperative BCVA between the unilateral and bilateral incision groups.

Parameters	Unilateral Incision	Bilateral Incision	*p*-Value
Preoperative			
Mean ± SD, LogMAR	−0.02 ± 0.10	0.03 ± 0.22	0.21
95% CI	−0.05, 0.01	−0.03, 0.10	
POW2			
Mean ± SD	−0.00 ± 0.11	0.05 ± 0.27	0.29
95% CI	−0.04, 0.04	−0.02, 0.13	
POM1			
Mean ± SD	−0.03 ± 0.08	0.02 ± 0.17	0.21
95% CI	−0.06, 0.01	−0.03, 0.06	
POM3			
Mean ± SD	−0.03 ± 0.08	−0.00 ± 0.12	0.40
95% CI	−0.06, 0.01	−0.04, 0.03	
POM6			
Mean ± SD	−0.02 ± 0.08	−0.01 ± 0.13	0.57
95% CI	−0.06, 0.01	−0.05, 0.03	
POM9			
Mean ± SD	−0.03 ± 0.09	0.00 ± 0.15	0.31
95% CI	−0.07, 0.01	−0.04, 0.04	
POM12			
Mean ± SD	−0.03 ± 0.07	−0.01 ± 0.13	0.37
95% CI	−0.06, −0.00	−0.04, 0.03	

*p*-values are calculated using an unpaired *t*-test. Abbreviations: BCVA—best-corrected visual acuity; LogMAR—logarithm of the minimum angle of resolution; SD—standard deviation; CI—confidence interval; POW—postoperative week; POM—postoperative month.

**Table 8:** Comparison of preoperative and postoperative ac flare between the unilateral and bilateral incision groups.

Parameters	Unilateral Incision	Bilateral Incision	*p*-Value
Preoperative			
Mean ± SD, pc/ms	9.7 ± 9.3	8.1 ± 4.6	0.31
95% CI	6.1, 13.2	6.8, 9.4	
POW2			
Mean ± SD	9.7 ± 5.3	13.8 ± 19.6	0.28
95% CI	7.7, 11.7	8.2, 19.3	
POM1			
Mean ± SD	9.2 ± 5.2	10.3 ± 5.7	0.37
95% CI	7.2, 11.1	8.7, 11.9	
POM3			
Mean ± SD	7.6 ± 2.8	9.5 ± 5.4	0.072
95% CI	6.5, 8.6	8.0, 11.0	
POM6			
Mean ± SD	7.5 ± 2.4	7.8 ± 2.7	0.61
95% CI	6.5, 8.4	7.0, 8.5	
POM9			
Mean ± SD	7.7 ± 2.2	8.7 ± 3.3	0.15
95% CI	6.8, 8.6	7.8, 9.7	
POM12			
Mean ± SD	8.0 ± 2.1	8.5 ± 3.7	0.52
95% CI	7.1, 8.9	7.5, 8.9	

*p*-values are calculated using an unpaired *t*-test. Abbreviations: AC—anterior chamber; pc/ms—photon counts per millisecond; SD—standard deviation; CI—confidence interval; POW—postoperative week; POM—postoperative month.

**Table 9:** Comparison of preoperative and postoperative CECD between the unilateral and bilateral incision groups.

Parameters	Unilateral Incision	Bilateral Incision	*p*-Value
Preoperative			
Mean ± SD, cells/mm^2^	2601 ± 268	2572 ± 213	0.59
95% CI	2499, 2703	2512, 2631	
POM6			
Mean ± SD	2548 ± 181	2550 ± 214	0.97
95% CI	2476, 2619	2489, 2610	
POM12			
Mean ± SD	2512 ± 185	2553 ± 229	0.44
95% CI	2434, 2590	2487, 2619	

*p*-values are calculated using an unpaired *t*-test. Abbreviations: CECD—corneal endothelial cell density; SD—standard deviation; CI—confidence interval; POM—postoperative month.

**Table 10:** Comparison of preoperative and postoperative MD between the unilateral and bilateral incision groups.

Parameters	Unilateral Incision	Bilateral Incision	*p*-Value
Preoperative			
Mean ± SD, dB	−9.6 ± 8.2	−8.7 ± 7.3	0.61
95% CI	−6.5, −12.7	−6.6, −10.7	
POM12			
Mean ± SD	−9.3 ± 7.3	−9.1 ± 6.9	0.94
95% CI	−12.4, −6.2	−11.1, −7.1	

*p*-values are calculated using an unpaired *t*-test. Abbreviations: MD—mean deviation; dB—decibel; SD—standard deviation; CI—confidence interval; POM—postoperative month.

**Table 11:** Possible factors associated with IOP control rate.

Parameters	HR	95% CI Range	*p*-Value
**Criteria A (Success: ≤18 mmHg and ≥20% reduction)**			
Age,/yr	0.99	0.97, 1.02	0.59
Sex, F/M	1.57	0.92, 2.69	0.098
JOAG/POAG	1.19	0.60, 2.38	0.62
Unilateral/Bilateral	0.98	0.53, 1.80	0.95
Preoperative IOP,/mmHg	0.98	0.94, 1.02	0.39
Preoperative Medication,/medication	1.12	0.85, 1.50	0.42
Surgical complication, y/n	0.73	0.42, 1.28	0.28
**Criteria B (Success: ≤15 mmHg and ≥20% reduction)**			
Age,/yr	1.00	0.97, 1.02	0.93
Sex, F/M	1.61	0.97, 2.67	0.066
JOAG/POAG	1.00	0.53, 1.88	>0.99
Unilateral/Bilateral	1.02	0.57, 1.82	0.95
Preoperative IOP,/mmHg	1.02	0.98, 1.05	0.27
Preoperative Medication,/medication	1.22	0.94, 1.61	0.14
Surgical complication, y/n	0.76	0.45, 1.31	0.33
**Criteria C (Success: ≤12 mmHg and ≥20% reduction)**			
Age,/yr	1.00	0.98, 1.03	0.74
Sex, F/M	1.14	0.71, 1.84	0.58
JOAG/POAG	0.83	0.47, 1.47	0.52
Unilateral/Bilateral	1.13	0.66, 1.94	0.65
Preoperative IOP,/mmHg	1.01	0.98, 1.04	0.51
Preoperative Medication,/medication	0.96	0.75, 1.25	0.78
Surgical complication, y/n	0.83	0.47, 1.47	0.46
**Criteria D (Success: ≤18 mmHg and ≥0% reduction)**			
Age,/yr	0.97	0.94, 1.01	0.12
Sex, F/M	1.66	0.84, 3.27	0.14
JOAG/POAG	1.13	0.53, 2.39	0.75
Unilateral/Bilateral	0.87	0.43, 1.78	0.70
Preoperative IOP,/mmHg	1.02	0.98, 1.06	0.28
Preoperative Medication,/medication	0.99	0.72, 1.39	0.96
Surgical complication, y/n	0.50	0.25, 0.99	0.048 *
**Criteria E (Success: ≤15 mmHg and ≥0% reduction)**			
Age,/yr	0.99	0.97, 1.02	0.46
Sex, F/M	1.60	0.93, 2.75	0.089
JOAG/POAG	0.83	0.43, 1.58	0.56
Unilateral/Bilateral	0.90	0.50, 1.64	0.74
Preoperative IOP,/mmHg	1.04	1.00, 1.07	0.028 *
Preoperative Medication,/medication	1.10	0.84, 1.45	0.49
Surgical complication, y/n	0.67	0.38, 1.17	0.16
**Criteria F (Success: ≤12 mmHg and ≥0% reduction)**			
Age,/yr	1.00	0.98, 1.03	0.74
Sex, F/M	1.14	0.71, 1.84	0.58
JOAG/POAG	0.83	0.47, 1.47	0.52
Unilateral/Bilateral	1.13	0.66, 1.94	0.65
Preoperative IOP,/mmHg	1.01	0.98, 1.04	0.51
Preoperative Medication,/medication	0.96	0.75, 1.25	0.78
Surgical complication, y/n	0.83	0.50, 1.37	0.46

*p*-values are calculated using the Cox hazard model based on six failure criteria. Criterion A defines failure as an IOP reduction of less than 20% from preoperative levels and/or an IOP exceeding 18 mmHg, while Criterion B applies the same percentage reduction threshold with an upper IOP limit of 15 mmHg, and Criterion C lowers this threshold to 12 mmHg. Criterion D considers failure to have occurred when the IOP reduction is less than 0% from preoperative levels and/or exceeds 18 mmHg, with Criterion E setting this limit at 15 mmHg and Criterion F at 12 mmHg. Patients who required additional glaucoma surgery or experienced a loss of light perception were also classified as failures. The * indicates *p* < 0.05. Abbreviations: HR—hazard ratio; F—female; M—male; JOAG—juvenile open-angle glaucoma; POAG—primary open-angle glaucoma; IOP—intraocular pressure.

## Data Availability

Data are fully available upon reasonable request to the corresponding author.

## References

[1] Heijl A. (2015). Glaucoma treatment: By the highest level of evidence. Lancet.

[2] Jayaram H., Kolko M., Friedman D.S., Gazzard G. (2023). Glaucoma: Now and beyond. Lancet.

[3] Ahmed I.I. (2015). MIGS and the FDA: What’s in a Name?. Ophthalmology.

[4] Chihara E., Hamanaka T. (2024). Historical and Contemporary Debates in Schlemm’s Canal-Based MIGS. J. Clin. Med..

[5] Gutkind N.E., Gedde S.J. (2025). Reporting outcomes of minimally invasive glaucoma surgery. Curr. Opin. Ophthalmol..

[6] Tanito M., Sugihara K., Tsutsui A., Hara K., Manabe K., Matsuoka Y. (2021). Midterm Results of Microhook ab Interno Trabeculotomy in Initial 560 Eyes with Glaucoma. J. Clin. Med..

[7] Rosenquist R., Epstein D., Melamed S., Johnson M., Grant W.M. (1989). Outflow resistance of enucleated human eyes at two different perfusion pressures and different extents of trabeculotomy. Curr. Eye Res..

[8] Hirabayashi M.T., Lee D., King J.T., Thomsen S., An J.A. (2019). Comparison of Surgical Outcomes of 360° Circumferential Trabeculotomy Versus Sectoral Excisional Goniotomy With The Kahook Dual Blade At 6 Months. Clin. Ophthalmol..

[9] Qiao Y., Tan C., Chen X., Sun X., Chen J. (2021). Gonioscopy-assisted transluminal trabeculotomy versus goniotomy with Kahook dual blade in patients with uncontrolled juvenile open-angle glaucoma: A retrospective study. BMC Ophthalmol..

[10] Yokoyama H., Takata M., Gomi F. (2022). One-year outcomes of microhook trabeculotomy versus suture trabeculotomy ab interno. Graefes Arch. Clin. Exp. Ophthalmol..

[11] Manabe S.I., Sawaguchi S., Hayashi K. (2017). The effect of the extent of the incision in the Schlemm canal on the surgical outcomes of suture trabeculotomy for open-angle glaucoma. Jpn. J. Ophthalmol..

[12] Sato T., Kawaji T. (2021). 12-month randomised trial of 360° and 180° Schlemm’s canal incisions in suture trabeculotomy ab interno for open-angle glaucoma. Br. J. Ophthalmol..

[13] Okada N., Hirooka K., Onoe H., Murakami Y., Okumichi H., Kiuchi Y. (2021). Comparison of Efficacy between 120° and 180° Schlemm’s Canal Incision Microhook Ab Interno Trabeculotomy. J. Clin. Med..

[14] Mori S., Murai Y., Ueda K., Sakamoto M., Kurimoto T., Yamada-Nakanishi Y., Nakamura M. (2021). Comparison of efficacy and early surgery-related complications between one-quadrant and two-quadrant microhook ab interno trabeculotomy: A propensity score matched study. Acta Ophthalmol..

[15] Song Y., Zhu X., Zhang Y., Shu J., Dang G., Zhou W., Sun L., Li F., Lin F., Zhang Y. (2023). Outcomes of Partial Versus Complete Goniotomy With or Without Phacoemulsification for Primary Open Angle Glaucoma: A Multicenter Study. J. Glaucoma.

[16] Zhang Y., Yu P., Zhang Y., Sugihara K., Zhu X., Zhang Y., Yang X., Li X., Liu Y., Zhang H. (2023). Influence of Goniotomy Size on Treatment Safety and Efficacy for Primary Open-Angle Glaucoma: A Multicenter Study. Am. J. Ophthalmol..

[17] Parikh H.A., Bussel I.I., Schuman J.S., Brown E.N., Loewen N.A. (2016). Coarsened Exact Matching of Phaco-Trabectome to Trabectome in Phakic Patients: Lack of Additional Pressure Reduction from Phacoemulsification. PLoS ONE.

[18] Neiweem A.E., Bussel I.I., Schuman J.S., Brown E.N., Loewen N.A. (2016). Glaucoma Surgery Calculator: Limited Additive Effect of Phacoemulsification on Intraocular Pressure in Ab Interno Trabeculectomy. PLoS ONE.

[19] Murata N., Takahashi E., Saruwatari J., Kojima S., Inoue T. (2023). Outcomes and risk factors for ab interno trabeculotomy with a Kahook Dual Blade. Graefes Arch. Clin. Exp. Ophthalmol..

[20] Sugihara K., Shimada A., Ichioka S., Harano A., Tanito M. (2023). Comparison of Phaco-Tanito Microhook Trabeculotomy between Propensity-Score-Matched 120-Degree and 240-Degree Incision Groups. J. Clin. Med..

[21] Benekos K., Katsanos A., Laspas P., Vagiakis I., Haidich A.B., Konstas A.G. (2024). Intraocular Pressure Reduction Following Phacoemulsification in Patients with Exfoliation: A Systematic Review and Meta-Analysis. J. Clin. Med..

[22] Bidiwala S., Jabarkhyl D., Bidiwala K. (2025). Outcomes of Minimally Invasive Glaucoma Surgery (MIGS) in Glaucoma Patients with Coexisting Cataract: A Systematic Review and Meta-Analysis. Cureus.

[23] Chan N.S., Sng C.C.A. (2025). Minimally invasive glaucoma surgery in angle closure. Curr. Opin. Ophthalmol..

[24] Torbey J., Mansouri K. (2025). Cataract surgery combined with glaucoma surgery. Curr. Opin. Ophthalmol..

[25] Yuan P.H.S., Dorling M., Shah M., Panarelli J.F., Durr G.M. (2025). Combined Microinvasive Glaucoma Surgery with Phacoemulsification in Open-Angle Glaucoma: A Systematic Review and Meta-analysis. Am. J. Ophthalmol..

[26] Grover S., Fishman G.A., Anderson R.J., Tozatti M.S., Heckenlively J.R., Weleber R.G., Edwards A.O., Brown J. (1999). Visual acuity impairment in patients with retinitis pigmentosa at age 45 years or older. Ophthalmology.

[27] Ishida A., Ichioka S., Takayanagi Y., Tsutsui A., Manabe K., Tanito M. (2021). Comparison of Postoperative Hyphemas between Microhook Ab Interno Trabeculotomy and iStent Using a New Hyphema Scoring System. J. Clin. Med..

[28] Tanito M., Sugihara K., Tsutsui A., Hara K., Manabe K., Matsuoka Y. (2021). Effects of Preoperative Intraocular Pressure Level on Surgical Results of Microhook Ab Interno Trabeculotomy. J. Clin. Med..

[29] Wecker T., Neuburger M., Bryniok L., Bruder K., Luebke J., Anton A., Jordan J.F. (2016). Ab Interno Trabeculectomy with the Trabectome as a Valuable Therapeutic Option for Failed Filtering Blebs. J. Glaucoma.

[30] Harano A., Shimada A., Ichioka S., Sugihara K., Tanito M. (2023). Fellow-Eye Comparison between Phaco-Tanito Microhook Trabeculotomy and Phaco-iStent Inject, W. J. Clin. Med..

[31] Chihara E., Chihara T. (2024). Consequences of Clot Formation and Hyphema Post-Internal Trabeculotomy for Glaucoma. J. Glaucoma.

